# A multi-pronged approach to improve blood culture diagnostics in different clinical departments: a single-centre experience

**DOI:** 10.1007/s15010-023-02083-y

**Published:** 2023-08-17

**Authors:** Elisabeth Neser, Philipp Jung, Alexander Halfmann, Matthias Schröder, Lorenz Thurner, Sören L. Becker, Sophie Schneitler

**Affiliations:** 1 Institute of Medical Microbiology and Hygiene, Saarland University, Kirrberger Strasse, Building 43, 66421 Homburg/Saar, Germany; 2 Clinic for Pneumology and Allergology, Bethanien Hospital, Centre of Sleep Medicine and Respiratory Care, Institute of Pneumology at the University of Cologne, Solingen, Germany; 3 Swiss Tropical and Public Health Institute, Allschwil, Switzerland; 4 University of Basel, Basel, Switzerland; 5 Department of Anaesthesiology, Intensive Care and Analgesic Therapy, Saarland University, Homburg/Saar, Germany; 6 Department of Internal Medicine Oncology, Haematology, Clinical Immunology and Rheumatology, Saarland University, Homburg/Saar, Germany

**Keywords:** Blood culture, Blood culture contamination, Diagnostic stewardship, Interdisciplinary medical education

## Abstract

**Purpose:**

Blood culture (BC) diagnostics are influenced by many factors. We performed a targeted interdisciplinary analysis to analyse effects of various measures on BC diagnostics performance.

**Methods:**

A diagnostic stewardship initiative was conducted at two intervention and two control wards in a German tertiary level hospital. The initiative comprised staff training on the correct indications and sampling for BC, implementation of information cards, labels to identify the collection site, regular BC bottle feedback including the number of bottles, filling volumes and identified pathogens; and the use of a specific sampling device (BD Vacutainer^®^). Before and after the interventions, two three-month measurement periods were performed, as well as a one-month follow-up period to assess the sustainability of the conducted measures.

**Results:**

In total, 9362 BC bottles from 787 patients were included in the analysis. The number of BCs obtained from peripheral venous puncture could be increased at both intervention wards (44.0 vs*.* 22.2%, 58.3 vs*.* 34.4%), while arterial sampling could be reduced (30.6 vs*.* 4.9%). A total of 134 staff members were fully trained. The intervention led to a considerable increase in BC knowledge (from 62.4 to 79.8% correct answers) with differences between the individual professional groups. Relevant reduced contamination rates could be detected at both intervention wards.

**Conclusions:**

As knowledge on the correct BC sampling and strategies to reduce contamination varies considerably between clinical departments and healthcare professionals, a targeted training should be adapted to the specific needs of the individual professional groups. An additional filling device is not necessary.

**Supplementary Information:**

The online version contains supplementary material available at 10.1007/s15010-023-02083-y.

## Introduction

Bloodstream infections are associated with high morbidity and mortality, and at least 5.3 million deaths per year are attributed to bacteraemia and sepsis worldwide [10]. Therefore, it is essential to detect these infections as early as possible, currently with the diagnostics of blood cultures (BCs). Rapid pathogen identification enables effective and targeted antibiotic therapy and can significantly improve patient outcomes [2]. However, this requires the attention to several pre-analytical aspects among others to achieve low contamination rate of the BCs, which should be less than 3%, to avoid unnecessary therapies, associated costs and prolonged hospital stays [17, 25].

A key aspect is the collection of a sufficient number of BCs to reduce false-negative and false-positive BCs, which can lead to incorrect clinical interpretations [1]. Thus, with the collection of three BC sets, one aerobic and one anaerobic bottle each, the sensitivity can be increased from 67.4% with one set to 95.6%. [25].

Insufficiently filled BCs are a frequent problem, connected to false-negative findings or a higher contamination rate, optimising the blood volume increases the sensitivity [3, 11, 16]. Therefore, filling volumes should be given a high priority.

BCs collected from catheters are more often false-positive, especially from arterial catheters [5, 15, 18, 19, 22]. Improvement is possible with peripherally sampled BCs, and the often less cumbersome catheter method should only be performed if a catheter infection is suspected. Educational interventions have already been successfully used in individual professional groups to improve BC diagnostics, however, the focus was not on interdisciplinary groups [2, 13, 20]. Therefore, this study focuses on the educational intervention in an interdisciplinary team with heterogeneous levels of training. This reflects and evaluates the common clinical practice. The intervention was therefore combined with a detailed staff survey to evaluate interdisciplinary knowledge and experience with BC diagnostics.

## Methods

### Study design

**Fig. 1:**
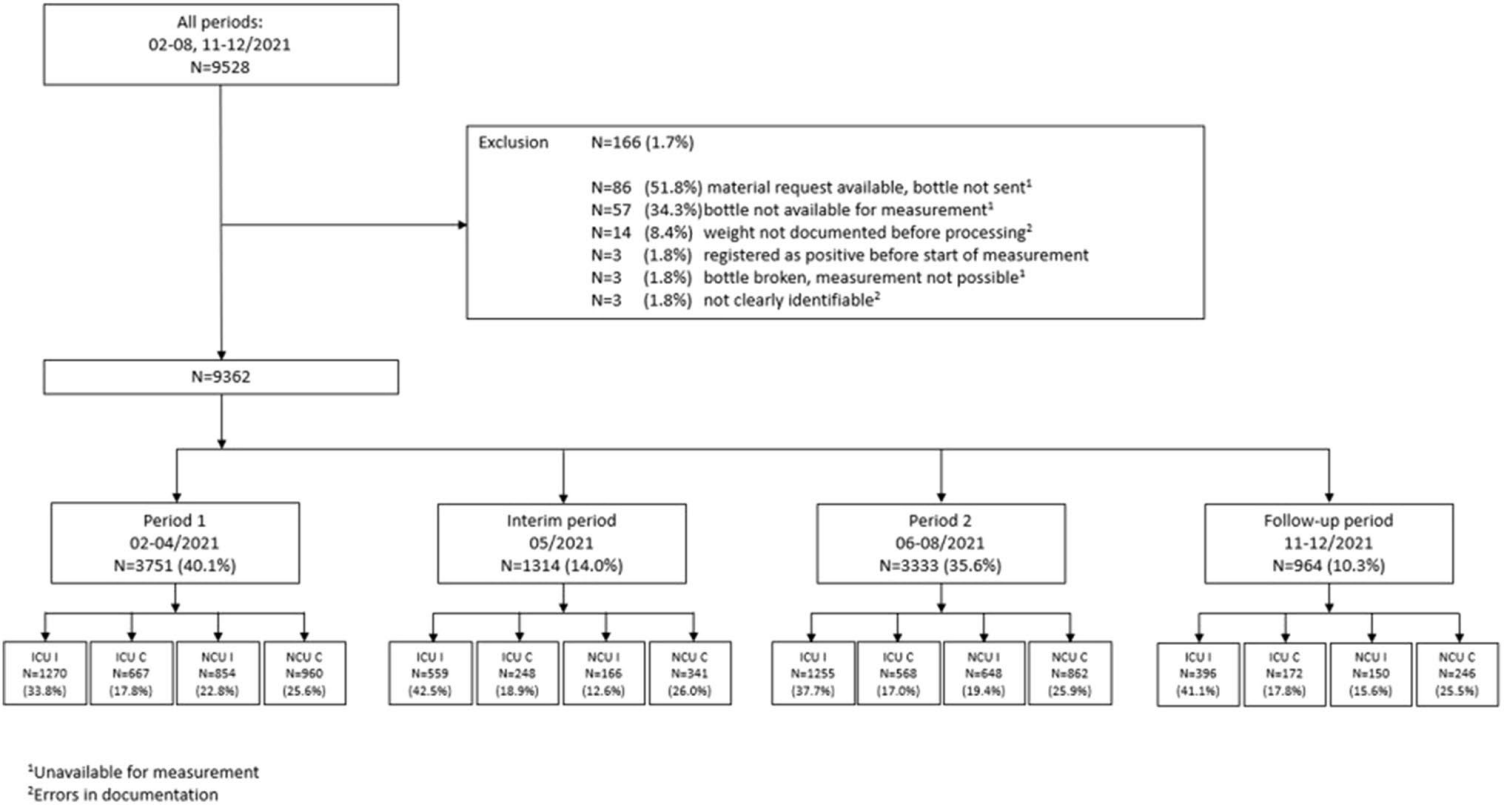
Flowchart of a study pertaining to improved blood culture diagnostics at a university hospital in Southwest Germany, February to December 2021. Overview of all time periods and exclusion

An intervention study was carried out on four wards at Saarland University Medical Center in Homburg, Germany, and the study comprised four different phases: From February to April (period 1; measurement), in May (interim period; Diagnostic Stewardship intervention including staff questioning, training, and implementation of different tools, continued measurement), from June to August (period 2; measurement), and from November to December 2021 (follow-up period; measurement). BC bottles were analysed by bottle weight and pathogen identification. A haematological normal care unit (NCU I) with monitoring facilities and a surgical intensive care unit (ICU I), each with matched control wards (NCU C, ICU C), selected on the basis of previous year's data regarding the total amount of BCs collected and potential contaminations (Fig. 1). A previously determined average weight of the empty bottles was used to calculate the blood volume they contained.

### Processing of the blood culture bottles

Aerobic, anaerobic, and Mycosis BC bottles of BD BACTEC™ (Becton, Dickinson and Company, Heidelberg, Germany) were used. The minimum incubation time was six days (35 ± 1 °C). In case of suspected cardiac infection an incubation of 14 days was conducted. Pathogen growth was detected in BACTEC FX (Becton, Dickinson and Company, Heidelberg, Germany) and identified according to internal standards by Gram staining and MALDI-TOF-based identification. New pathogens were reported by telephone to the responsible physicians. Likely contaminants, i.e. coagulase-negative staphylococci (CONS) or *Cutibacterium* spp. were interpreted using clinical information [4, 12].

### Staff questioning, training and implementation of new diagnostic tools

Interdisciplinary training sessions à 15–20 min were conducted mainly during handovers (optional digital use of recorded trainings), aiming at providing knowledge, presenting new measures, and generally sensitising the staff to the topic of BC diagnostics.

Anonymised participant questionnaires with ten multiple-select questions were distributed before and after the training, containing general respondents’ information and BC-knowledge (supplementary material).

The implemented tools included BC pocket sized information cards, new BC labelling (supplementary material), weekly feedback to ward managers on the BC statistic including numbers, pathogens, contamination status and calibration marks. The BD Vacutainer^®^ blood collection set, a winged cannula with attached vacuum adapter, was launched in ICU I.

After period 2, the staff questionnaire was used again with new questions related to the tools.

Analysis and calculations were subsequently carried out using Microsoft^®^ Excel^®^ (Microsoft 365, Redmond, United States of America) and GraphPad Prism (GraphPad Software, San Diego, United States of America).

## Results

### Baseline characteristics

**Fig. 2:**
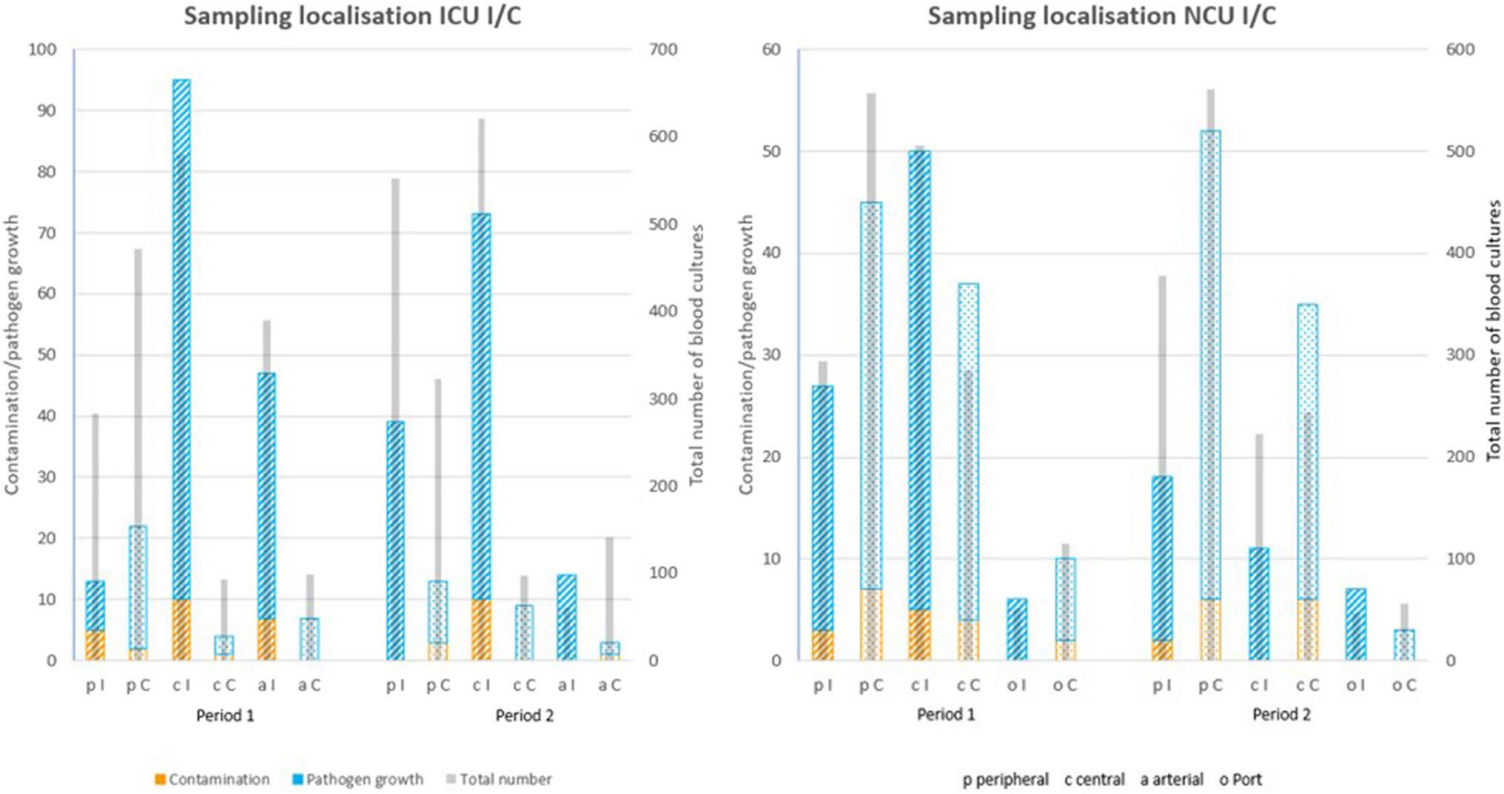
Sampling localisations in a study pertaining to improved blood culture diagnostics at a university hospital in southwest Germany, February to December 2021. Pathogen growth and contamination depending on sampling localisation. Intervention wards (I) are striped, control wards (C) are dotted. Blue and orange bars refer to the left axis, the grey ones to the right axis. *p* peripheral, *c* central, *a* arterial, *o* port catheter

During the study period, 9362 BC bottles from 787 patients were included, of which 3268 (34.9%) were aerobic, 3300 (35.2%) anaerobic and 2792 (29.8%) Mycosis (fungal) bottles. A subset of 165 (1.7%) BCs were excluded, mostly due to unavailability for weight measurement (87.9%), which occurred for example, when a material request for BC was received but the bottle was not sent in, or when the bottle was broken (Fig. 1). In 780 (8.3%) bottles, pathogens could be detected, 109 (14.0%) of these were interpreted as likely contamination (Fig. 2).

The average age of the patients was similar on all wards (63.9 years to 69.6 years). Only in NCU I patients were around 10 years younger. At all wards, more male patients (64.3%) were treated than female patients (35.7%). The intervention wards (IWs) had a median duration of stay over 20 days (Table 1).

**Table 1:** General information

Patient-related data	Period 1				Interim period				Period 2				Follow-up period			
	ICU I (N = 126)	ICU C (N = 51)	NCU I (N = 42)	NCU C (N = 87)	ICU I (N = 56)	ICU C (N = 15)	NCU I (N = 20)	NCU C (N = 36)	ICU I (N = 100)	ICU C (N = 39)	NCU I (N = 44)	NCU C (N = 73)	ICU I (N = 35)	ICU C (N = 10)	NCU I (N = 20)	NCU C (N = 33)
Average age (years)	65.5	64.7	55.2	64.7	66.3	65.8	60.4	64	63.9	65	53.5	63.6	66.9	69.6	58.8	67.2
Male (%)	79 (62.7)	41 (80.4)	26 (61.9)	56 (64.4)	36 (64.3)	12 (80)	10 (50)	20 (55.6)	64 (64)	26 (66.7)	21 (47.7)	45 (61.6)	22 (62.9)	9 (90)	16 (80)	23 (69.7)
Female (%)	47 (37.3)	10 (19.6)	16 (38.1)	32 (36.8)	20 (35.7)	3 (20)	10 (50)	16 (44.4)	36 (36)	13 (33.3)	22 (50)	28 (38.4)	13 (37.1)	1 (10)	4 (20)	10 (30.3)
Median duration of stay (days)	20.9	17.1	24.2	14	18.6	23.1	25.6	14.4	24	17.5	22.1	12.3	31.9	14.3	25.1	16.6
Median Leukocytes (10\S\9/l)	11.4	12.9	0.3	3.3	13.95	14.5	0.85	1.5	12.1	12.15	0.9	4.1	12.4	14.5	0.3	4.9
Median CRP (mg/l)	130.7	110.2	83.9	87.2	125.5	81.8	81.9	91	124.7	75.1	63.7	75.5	101.9	123.5	73.1	80.3

Stay-related data	ICU I (N = 129)	ICU C (N = 51)	NCU I (N = 55)	NCU C (N = 103)	ICU I (N = 56)	ICU C (N = 15)	NCU I (N = 20)	NCU C (N = 36)	ICU I (N = 101)	ICU C (N = 40)	NCU I (N = 58)	NCU C (N = 88)	ICU I (N = 35)	ICU C (N = 10)	NCU I (N = 20)	NCU C (N = 34)
Stays with positive BC (%)	51 (39.5)	17 (33.3)	16 (29.1)	29 (28.2)	21 (37.5)	8 (53.3)	5 (25)	10 (27.8)	35 (34.7)	15 (37.5)	15 (25.9)	25 (28.4)	16 (45.71)	3 (30)	4 (20)	9 (26.5)
Average number of positive episodes	0.6	0.5	0.6	0.3	1.9	1.5	2.2	1.7	1.9	1.8	1.5	1.4	1.6	2.7	2.5	1
Outcome dismissed (%)	67 (51.9)	35 (68.6)	49 (89.1)	83 (80.6)	29 (51.8)	9 (60)	17 (85)	30 (83.3)	53 (52.5)	19 (47.5)	55 (94.8)	78 (88.6)	19 (54.3)	4 (40)	18 (90)	28 (82.4)
With positive BC	35 (68.6)	7 (41.2)	13 (81.3)	20 (69)	11 (52.4)	6 (75)	4 (80)	8 (80)	18 (51.4)	5 (33.3)	13 (86.7)	21 (84)	6 (37.5)	1 (33.3)	3 (75)	7 (77.8)
Transferred to external hospital	23 (17.8)	10 (19.6)	0	1 (1.0)	8 (14.3)	3 (20)	0	0	21 (20.8)	16 (40)	1 (1.7)	0	2 (5.71)	3 (30)	0	1 (2.9)
With positive BC	8 (15.7)	6 (35.3)	0	1 (3.5)	2 (9.5)	1 (12.5)	0	0	9 (25.7)	8 (53.3)	0	0	1 (6.25)	1 (33.3)	0	0
Deceased	39 (30.2)	6 (11.8)	6 (10.9)	19 (18.5)	19 (33.9)	3 (20)	3 (15)	5 (13.9)	27 (36.7)	5 (12.5)	2 (3.5)	10 (11.4)	14 (40)	3 (30)	2 (10)	5 (14.7)
With positive BC	18 (35.3)	4 (23.5)	3 (18.8)	8 (27.6)	8 (38.1)	1 (12.5)	1 (20)	2 (20)	8 (22.9)	2 (13.3)	1 (6.7)	4 (16)	7 (43.75)	1 (33.3)	0	2 (22.2)

General information on patients’ characteristics in a study pertaining to improved blood culture diagnostics at a university hospital in Southwest Germany, February to December 2021

### Distribution of microbiological pathogens

CONS were detected with the highest rate (from 45.4 to 74.0% of all pathogens). Gram-negative pathogens could be detected with a rate of 24.6 to 30.5% (commonest *Escherichia coli*), except on ICU C (Table 2).

**Table 2:** Detected pathogens in a study pertaining to improved blood culture diagnostics at a university hospital in Southwest Germany, February to December 2021

	Total period				Period 1				Interim period				Period 2				Follow-up period			
	ICU I (N = 394)	ICU C (N = 73)	NCU I (N = 141)	NCU C (N = 208)	ICU I (N = 179)	ICU C (N = 31)	NCU I (N = 82)	NCU C (N = 87)	ICU I (N = 57)	ICU C (N = 16)	NCU I (N = 10)	NCU C (N = 33)	ICU I (N = 118)	ICU C (N = 21)	NCU I (N = 34)	NCU C (N = 82)	ICU I (N = 40)	ICU C (N = 5)	NCU I (N = 15)	NCU C (N = 8)
*Staphylococcus aureus* (%)	43 (10.9)	0	0	10 (4.8)	13 (7.3)	0	0	6 (6.9)	18 (31.6)	0	0	1 (3)	10 (8.5)	0	0	3 (3.7)	2 (5)	0	0	0
CONS	179 (45.4)	54 (74)	57 (40.4)	97 (46.6)	99 (55.3)	27 (87.1)	29 (35.4)	32 (36.8)	22 (38.6)	5 (31.3)	4 (40)	13 (39.4)	40 (33.9)	18 (85.7)	22 (64.7)	48 (58.5)	18 (45)	4 (80)	2 (13.3)	4 (50)
Streptococcus spp.	10 (2.5)	0	2 (1.4)	10 (4.8)	2 (1.1)	0	1 (1.22)	4 (4.6)	2 (3.5)	0	0	1 (3.0)	6 (5.1)	0	1 (2.9)	5 (6.1)	0	0	0	0
Enterococcus spp.	35 (8.9)	9 (12.3)	28 (19.9)	19 (9.1)	21 (11.7)	0	22 (26.8)	16 (18.4)	1 (1.8)	9 (56.3)	3 (30)	2 (6.1)	11 (9.3)	0	0	0	2 (5)	0	3 (20)	1 (12.5)
other Gram-positive pathogens	7 (1.8)	3 (4.1)	4 (2.8)	8 (3.8)	5 (2.8)	2 (6.5)	3 (3.7)	4 (4.6)	2 (3.5)	0	0	1 (3.0)	0	1 (4.8)	1 (2.9)	3 (3.7)	0	0	0	0
Gram-negative pathogens	97 (24.6)	0	43 (30.5)	56 (26.9)	29 (16.2)	0	23 (28.1)	19 (21.8)	12 (21.1)	0	3 (30)	14 (42.4)	43 (36.4)	0	8 (23.5)	22 (26.8)	13 (32.5)	0	9 (60)	1 (12.5)
*Candida* spp.	23 (5.8)	7 (9.6)	7 (5)	8 (3.9)	10 (5.6)	2 (6.5)	4 (4.9)	6 (6.9)	0	2 (12.5)	0	1 (3)	8 (6.8)	2 (9.5)	2 (5.9)	1 (1.2)	5 (12.5)	1 (20)	1 (6.7)	0

CONS: *Staphylococcus epidermidis*, *S. capitis*, *S. caprae*, *S. haemolyticus*, *S. hominis*, *S. pettenkoferi*, *S. saccharolyticus*. Other Gram-positive pathogens: *Aerococcus* spp., *Bacillus* spp., *Clostridium* spp., *Corynebacterium* spp., *Cutibacterium acnes*, *Gemella* spp., *Lactococcus* spp. Gram-negative pathogens: most frequently detected *Escherichia coli*, *Klebsiella* spp., *Pseudomonas* spp.

### Staff training and survey

Among the 134 trained persons (86.5% of the total staff), 77 (57.5%) were women. A total of 88 (82.2%) were trained in ICU I and 46 (95.8%) in NCU I. Main groups were the nursing staff [ICU I: 69 (78.4%), NCU I: 22 (47.8%)] and the physicians [ICU I: 19 (21.6%) NCU I: 17 (37.0%)]. There was no relevant change in the follow-up survey.

**Fig. 3:**
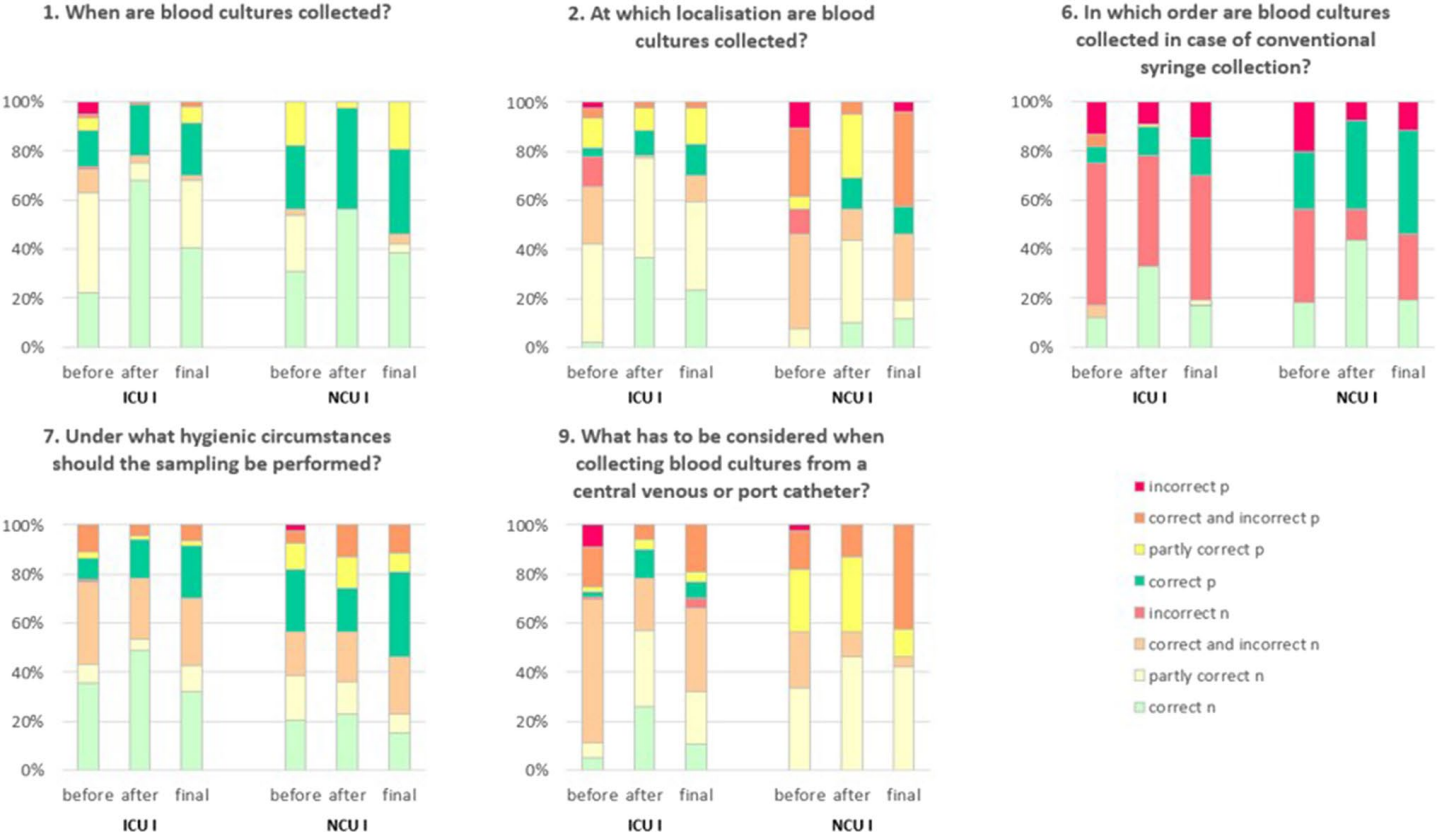
Answers of nursing staff and physicians to selected questions of the survey before and after the training as well as the final survey in a study pertaining to improved blood culture diagnostics at a university hospital in Southwest Germany, February to December 2021. Data in % for better comparison due to different number of participants by each survey period. *n* nursing staff, *p* physicians, incorrect: all answers are wrong, partly correct: not all correct answers were selected

Before the intervention, 32.7% of the knowledge questions were answered correctly or partially correctly (30.7%), i.e. not all correct answers were selected, but no incorrect ones either. After the training, there was a clear improvement to 57.8% correct and 22.0% partially correct answers. This effect reduced to 51.2% correct and 20.9% partially correct answers in the follow-up survey. Before the training, different answers in all areas of BC diagnostics between the professional groups were given. The question pertaining to BC indication was correctly answered in 30.0% by the nursing staff and 70.0% of the physicians in ICU I. Similar results were obtained regarding "hygiene and BC". Here 36.4% of the nursing staff had answered correctly and 58.8% of the physicians in NCU I (Table 3; Fig. 3). The handling of the BC indication was divergent among Intervention Ward’s (IWs). This, as well as the choice of sampling site, was made by physicians in 92.0% of cases in ICU I, while 37.6% of the NCU I respondents said that the nursing staff and physicians were responsible. The sampling itself was carried out by the entire staff on both wards, on NCU I also by the medical assistants.

**Table 3:** Staff survey in a study pertaining to improved blood culture diagnostics at a university hospital in Southwest Germany, February to December 2021

Questions	ICU I before training	ICU I after training	ICU I final questioning	NCU I before training	NCU I after training	NCU I final questioning
	N = 90 (%)	N = 88 (%)	N = 47 (%)	N = 46 (%)	N = 46 (%)	N = 26 (%)
Question 1: When are blood cultures collected?						
Correct total	35 (38.9)	78 (88.6)	29 (61.7)	26 (56.5)	44 (95.7)	19 (73.1)
Correct nursing staff	21 (30)	60 (87)	19 (57.6)	12 (54.6)	22 (100)	10 (83.3)
Correct physicians	14 (70)	18 (94.7)	10 (71.4)	10 (58.8)	16 (94.1)	9 (64.3)
Partly correct total	44 (48.9)	6 (6.8)	16 (34)	19 (41.3)	2 (4.4)	6 (23.1)
Partly correct nursing staff	39 (55.7)	6 (8.7)	13 (39.4)	9 (40.9)	0	1 (8.3)
Partly correct physicians	5 (25)	0	3 (21.4)	7 (41.2)	1 (5.9)	5 (35.7)
Correct and incorrect total	10 (11.1)	4 (4.6)	2 (4.3)	1 (2.2)	0	1 (3.9)
Correct and incorrect nursing staff	9 (12.9)	3 (4.4)	1 (3)	1 (4.6)	0	1 (8.3)
Correct and incorrect physicians	1 (5)	1 (5.3)	1 (7.1)	0	0	0
Question 2: At which localisation are blood cultures collected?						
Correct total	5 (5.6)	41 (46.6)	17 (36.2)	0	11 (23.9)	6 (23.1)
Correct nursing staff	2 (2.9)	32 (46.4)	11 (33.3)	0	4 (18.2)	3 (25)
Correct physicians	3 (15)	9 (47.4)	6 (42.9)	0	5 (29.4)	3 (21.4)
Partly correct total	47 (52.2)	44 (50)	24 (51.1)	7 (15.2)	28 (60.9)	2 (7.7)
Partly correct nursing staff	36 (51.4)	36 (52.2)	17 (51.5)	3 (13.6)	13 (59.1)	2 (16.7)
Partly correct physicians	11 (55)	8 (42.1)	7 (50)	2 (11.8)	10 (58.8)	0
Correct and incorrect total	25 (27.8)	3 (3.4)	6 (12.8)	30 (65.2)	7 (15.2)	17 (65.4)
Correct and incorrect nursing staff	21 (30)	1 (1.5)	5 (15.2)	15 (68.2)	5 (22.7)	7 (58.33
Correct and incorrect physicians	4 (20)	2 (10.5)	1 (7.1)	11 (64.7)	2 (11.8)	10 (71.4)
Incorrect total	13 (14.4)	0	0	9 (19.6)	0	1 (3.9)
Incorrect nursing staff	11 (15.7)	0	0	4 (18.2)	0	0
Incorrect physicians	2 (10)	0	0	4 (23.5)	0	1 (7.1)
Question 3: How many blood cultures should be collected at what interval?						
Correct total	11 (12.2)	25 (28.4)	13 (27.7)	10 (21.7)	23 (50)	13 (50)
Correct nursing staff	1 (1.4)	11 (15.9)	4 (12.1)	2 (9.1)	11 (50)	7 (58.3)
Correct physicians	10 (50)	14 (73.7)	9 (64.3)	7 (41.2)	3 (17.7)	6 (42.9)
Partly correct total	26 (28.9)	42 (47.7)	26 (55.3)	21 (45.7)	22 (47.8)	12 (46.2)
Partly correct nursing staff	18 (25.7)	40 (58)	21 (63.6)	11 (50)	10 (45.5)	5 (41.7)
Partly correct physicians	8 (40)	2 (10.5)	5 (35.7)	6 (35.3)	9 (52.9)	7 (50)
Correct and incorrect total	6 (6.7)	5 (5.7)	0	5 (10.9)	1 (2.2)	0
Correct and incorrect nursing staff	5 (7.1)	4 (5.8)	0	4 (18.2)	1 (4.6)	0
Correct and incorrect physicians	1 (5)	1 (5.3)	0	1 (5.9)	0	0
Incorrect total	47 (52.2)	16 (18.2)	8 (17)	10 (21.7)	0	1 (3.9)
Incorrect nursing staff	46 (65.7)	14 (20.3)	8 (24.2)	5 (22.7)	0	0
Incorrect physicians	1 (5)	2 (10.5)	0	3 (17.7)	0	1 (7.1)
Question 4: How much blood should be filled in an aerobic or anaerobic blood culture?						
Correct total	87 (96.7)	87 (98.9)	45 (95.7)	34 (73.9)	45 (97.8)	24 (92.3)
Correct nursing staff	67 (95.7)	68 (98.6)	31 (93.9)	18 (81.8)	21 (95.5)	11 (91.7)
Correct physicians	20 (100)	19 (100)	14 (100)	12 (70.6)	17 (100)	13 (92.9)
Incorrect total	1 (1.1)	0	0	9 (19.6)	1 (2.2)	1 (3.9)
Incorrect nursing staff	1 (1.4)	0	0	4 (18.2)	1 (4.6)	0
Incorrect physicians	0	0	0	3 (17.7)	0	1 (7.1)
Question 5: What are different blood culture bottles used for?						
Correct total	69 (76.7)	64 (72.7)	35 (74.5)	21 (45.7)	29 (63)	16 (61.5)
Correct nursing staff	57 (81.4)	48 (54.5)	25 (75.8)	13 (59.1)	13 (59.2)	9 (75)
Correct physicians	12 (60)	16 (84.2)	10 (71.4)	7 (41.2)	11 (64.7)	7 (50)
Partly correct total	10 (11.1)	3 (3.4)	1 (2.1)	10 (21.7)	4 (8.7)	5 (19.2)
Partly correct nursing staff	8 (11.4)	2 (2.9)	1 (3)	3 (13.6)	1 (4.6)	1 (8.3)
Partly correct physicians	2 (10)	1 (5.3)	0	5 (29.4)	3 (17.7)	4 (28.6)
Correct and incorrect total	10 (11.1)	21 (23.9)	11 (23.4)	12 (26.1)	13 (28.3)	5 (19.2)
Correct and incorrect nursing staff	5 (7.1)	19 (27.5)	7 (21.2)	5 (22.7)	8 (36.4)	2 (16.7)
Correct and incorrect physicians	5 (25)	2 (10.5)	4 (28.6)	4 (23.5)	3 (17.7)	3 (21.4)
Question 6: In which order are blood cultures inoculated in case of conventional syringe collection?						
Correct total	19 (21.1)	39 (44.3)	15 (31.9)	18 (39.1)	37 (80.4)	16 (61.5)
Correct nursing staff	12 (17.1)	29 (42)	8 (24.2)	7 (31.8)	17 (77.3)	5 (41.7)
Correct physicians	7 (35)	10 (52.6)	7 (50)	9 (52.9)	14 (82.4)	11 (78.6)
Incorrect total	71 (78.9)	48 (54.6)	31 (66)	28 (60.9)	9 (19.6)	10 (38.5)
Incorrect nursing staff	58 (82.9)	40 (58)	24 (72.7)	15 (68.2)	5 (22.7)	7 (58.3)
Incorrect physicians	13 (65)	8 (42.1)	7 (50)	8 (47.1)	3 (17.7)	3 (21.4)
Question 7: Under what hygienic circumstances should the collection be performed?						
Correct total	40 (44.4)	57 (64.8)	25 (53.2)	23 (50)	20 (43.5)	13 (50)
Correct nursing staff	32 (45.7)	43 (62.3)	15 (45.5)	8 (36.4)	9 (40.9)	4 (33.3)
Correct physicians	8 (40)	14 (73.7)	10 (71.4)	10 (58.8)	7 (41.2)	9 (64.3)
Partly correct total	9 (10)	5 (5.7)	6 (12.8)	11 (23.9)	11 (23.9)	4 (15.4)
Partly correct nursing staff	7 (10)	4 (5.8)	5 (15.2)	7 (31.8)	5 (22.7)	2 (16.7)
Partly correct physicians	2 (10)	1 (5.3)	1 (7.1)	4 (23.5)	5 (29.4)	2 (14.3)
Correct and incorrect total	40 (44.4)	26 (29.6)	16 (34)	11 (23.9)	15 (32.6)	9 (34.6)
Correct and incorrect nursing staff	30 (42.9)	22 (31.9)	13 (39.4)	7 (31.8)	8 (36.4)	6 (50)
Correct and incorrect physicians	10 (50)	4 (21.1)	3 (21.4)	2 (11.8)	5 (29.4)	3 (21.4)
Question 8: What has to be considered when labelling the bottles?						
Correct total	17 (18.9)	70 (79.6)	39 (83)	7 (15.2)	20 (43.5)	18 (69.2)
Correct nursing staff	10 (14.3)	52 (75.4)	16 (48.5)	0	3 (13.6)	5 (41.7)
Correct physicians	7 (35)	18 (94.7)	13 (92.9)	6 (35.3)	12 (70.6)	13 (92.9)
Partly correct total	64 (71.1)	14 (15.9)	7 (14.9)	34 (73.9)	24 (52.2)	7 (26.9)
Partly correct nursing staff	51 (72.9)	13 (18.8)	6 (18.2)	19 (86.4)	18 (81.8)	7 (58.3)
Partly correct physicians	13 (65)	1 (5.3)	1 (7.1)	10 (58.8)	5 (29.4)	0
Question 9: What has to be considered when withdrawing blood cultures from central venous or port catheters?						
Correct total	7 (7.8)	33 (37.5)	8 (17)	0	2 (4.4)	0
Correct nursing staff	5 (7.1)	23 (33.3)	5 (15.1)	0	0	0
Correct physicians	2 (10)	10 (52.6)	3 (21.4)	0	0	0
Partly correct total	8 (8.9)	31 (35.2)	12 (25.5)	24 (52.2)	32 (69.6)	14 (53.9)
Partly correct nursing staff	6 (8.6)	27 (39.1)	10 (30.3)	13 (59.1)	18 (81.8)	11 (91.7)
Partly correct physicians	2 (10.0)	4 (21.1)	2 (14.3)	10 (58.8)	12 (70.6)	3 (21.4)
Correct and incorrect total	74 (82.2)	24 (27.3)	25 (53.2)	19 (41.3)	12 (26.1)	12 (46.2)
Correct and incorrect nursing staff	58 (82.9)	19 (27.5)	16 (48.5)	9 (40.9)	4 (18.2)	1 (8.3)
Correct and incorrect physicians	16 (80)	5 (26.3)	9 (64.3)	6 (35.3)	5 (29.4)	11 (78.6)
Question 10: What has to be considered after the blood culture collection?						
Correct total	4 (4.4)	32 (36.4)	13 (27.7)	3 (6.5)	18 (39.1)	10 (38.5)
Correct nursing staff	3 (4.3)	27 (39.1)	10 (30.3)	0	11 (50)	8 (66.7)
Correct physicians	1 (5)	5 (26.3)	3 (21.4)	3 (17.7)	5 (29.4)	2 (14.3)
Partly correct total	50 (55.6)	32 (36.4)	16 (34)	33 (71.7)	22 (47.8)	8 (30.8)
Partly correct nursing staff	41 (58.6)	23 (33.3)	11 (33.3)	20 (90.9)	10 (45.4)	3 (25)
Partly correct physicians	9 (45)	9 (47.4)	5 (35.7)	10 (58.8)	9 (52.9)	5 (35.7)
Correct and incorrect total	27 (30)	16 (18.2)	13 (27.7)	8 (17.4)	6 (13)	8 (30.8)
Correct and incorrect nursing staff	21 (30)	13 (18.8)	8 (24.2)	2 (9.1)	1 (4.6)	1 (8.3)
Correct and incorrect physicians	6 (30)	3 (15.8)	5 (35.7)	3 (17.7)	3 (17.7)	7 (50)

Correct: all correct answers ticked. Partly correct: not all correct answers ticked, but no incorrect ones either. Correct and incorrect: correct and incorrect answers ticked. Incorrect: no correct answers ticked

Tools were generally rated well, but with different rankings among IWs. In ICU I, staff training was rated highest, followed by new labelling of the collection sites and information cards. NCU I preferred the cards, followed by training and labels. This is confirmed, since the label use analysis was better accepted in ICU I, than in NCU I: 91.5 vs. 80.8%. The BD Vacutainer^®^ was only preferred by 6.4%.

### Sampling sites

At ICU I, most of BCs were drawn from central venous catheters in period 1 (45.7%), followed by arterial catheters (30.6%) and peripheral veins (22.2%). In comparison, more BCs were drawn from peripheral veins in period 2 (44.0%), while arterial ones were significantly less frequently (4.9%, p < 0.0001). Central venous collections remained on an equal level (p = 0.04) (Fig. 2).

At NCU I, more BCs were collected from central than peripheral veins in period 1 (59.1 vs*.* 34.4%), which was reversed in period 2 (p < 0.0001).

The contamination rate was reduced at both IWs in all sampling localisations (total reduction period 1 vs*.* period 2 of 4.9 to 2.1% at ICU I and 2.2 to 0.4% at NCU I) (Fig. 2).

### Blood culture bottle characteristics

Across all time periods, aerobic bottles were filled with 9.6 ml in ICU I and with 8.3 ml in NCU I. The same was true for standard anaerobic (9.5 vs*.* 8.2 ml), and anaerobic lytic (9.9 vs*.* 8.7 ml). Since the Mycosis bottles (MB) are available with different bottle materials, both batches were purchased on availability (56.1% glass and 43.9% plastic bottles) and resulted in divergent blood filling volumes of plastic (9.9 vs*.* 8.7 ml) and glass bottles (10.4 vs*.* 9.3 ml). Considering the study period, 17.1% of MB were underfilled and 42.2% were overfilled. Glass bottles were overfilled compared to plastic bottles (p = 0.0029). This was no longer detectable after the intervention (p = 0.55) on ICU I. Furthermore, the MB calibration mark to indicate the optimal filling level was applied in 29.0% of the cases on ICU I, compared to 0.0% of cases during period 1.

## Discussion

In recent years, most research was done to improve the analytical part of BC diagnostics [23]. BC diagnostics is one of the few infectious medicine procedures in which clinical staff decide on incubation media and influence the result. Pre-analytics are therefore of atmost importance. This intervention study describes a diagnostic stewardship initiative pertaining to BC diagnostics across different wards of a university hospital. It included knowledge evaluations, a staff-reported assessment of implemented tools and a laboratory-based appraisal of BC diagnostics. To our knowledge, such a combined approach has not previously been carried out.

### Microbiological pathogens

Improved BC diagnostics involves the evaluation of the pathogen spectrum. Here, CONS was the most detected group of pathogens. There is the need to evaluate between a catheter infection and a contamination, especially if only one BC out of several is reported positive [4, 12, 14, 27]. There was an overall decrease of contaminations and CONS (from 55.3 to 33.9%) at ICU I in interim period and period 2 (from 4.9 to 2.1%) with recommended contamination rates less than 3% of all BC sets sampled [7, 17]. Questions regarding hygiene and BC sampling were answered correctly more often after training. Similar effects were demonstrated in previous studies, where a lower proportion of CONS was detected after training [15, 16, 20, 21]. In contrast, at NCU I a less frequent detection of CONS was not seen, yet a general decrease in contaminations (from 2.2 to 0.4%), demonstrating good training effects. The comparison of ICU I and NCU I shows that sole use of the BD Vacutainer^®^ in ICU I produced decreasing contamination rates, but that training alone achieved similar results.

It is noteworthy that no Gram-negative pathogens were detected at ICU C. Hence, we compared the Gram-negative pathogens detected in previous years with the study time. We found several detections of Gram-negative pathogens in previous years, but still below the expected range, which suggests room for improvement pertaining to adequate blood culture sampling indication on this ward. Additionally, the specific patient population treated on ICU C may be less at risk for Gram-negative bacteraemia.

### Sampling sites

Closely related to contamination is the choice of BC collection localisation and contaminations appear more frequently when BCs are drawn from catheters [5, 15, 21]. The interpretation of a positive BC from this localisation is more complicated [9, 12, 24]. The decrease in contaminations is probably a changed behaviour of both IWs concerning sampling localisation after training. This is indicated by the evidence of significantly more correctly answered questions after training (57.8% before vs*.* 96.6% correct and partially correct answers after training on ICU I and 15.2% before vs*.* 84.8% on NCU I), while the control wards showed no changes. During the follow-up period, the contamination rate decreased in NCU I, but increased slightly in ICU I, highlighting the importance of training repetition to revise routinized procedures [26].

The study data indicate the relevance to know who is responsible for BC diagnstics. It was found that physicians and medical assistants perform peripheral BC, while nursing staff perform the catheter BCs. After training, there was evidence of increased peripheral collection, so it can be assumed that tasks were delegated less frequently even if catheters were available due to the new knowledge of BC diagnostics.

### Staff survey

The core intervention carried out was an interdisciplinary transfer of knowledge to nursing staff, medical assistants, and physicians of the IWs. To our knowledge, studies conducted so far have not differentiated between the individual professional groups within one study [6, 8].

The increase in knowledge was most pronounced among the nursing staff. Training in BC diagnostics is not part of the nursing curriculum in Germany and there is no compulsory training after graduation. This explains the lower initial level of correct answers before training, approaching those of physicians afterwards. This may be one reason for the improved contamination rate, as De Dios Garcia et al. showed an increase in knowledge after a nursing staff training, but the contamination rate could not be reduced. [6].

Prior to the training, there was a gap in knowledge especially regarding the correct labelling of BCs, their storage and logistics. This underlines the importance to ensure error-free pre-analytics. In general, based on the staff feedback and results the tools were well received. Differences became apparent in the weekly feedback. These were rated well when actively forwarded by the ward manager to the staff. The more frequently the tools were used, the better the rating. The BD Vacutainer® received critical feedback, with longer removal times, a more difficult technique and insufficient practicability being mentioned.

Correct BC collection requires a lot of prior knowledge, especially regarding the correct blood volume with marking of the filling volume to optimise BC diagnostics but also patient blood management. Overall, there are indications that the bottle design is not adapted to the advancing digitalisation of healthcare system. The feedback indicates an improvement potential through a predefined vacuum and optimised labelling for the BCs.

### Sub-analysis of Mycosis blood cultures

The Mycosis bottle with specially designed media composition can be an addition in special cases. However, it was found that this type of bottle is particularly dependent on the bottle material for the amount of blood inoculated. One reason for this could be that a different bottle weight or the slightly larger size of the glass MB could have led to a misguided haptic. In most cases, the calibration mark recommended by the manufacturer was not considered during filling, as was the case with the other types of bottles. Mycosis glass BCs were more likely to be overfilled. After staff training, the fill levels of all MB no longer differed, even though this was not explicitly addressed in the training. Since no more statistically significant difference in blood filling volume in relation to the bottle material could be detected after the training. The training seems to be sufficient to overcome such previously unknown peculiarities. However, a possible effect of material-dependent performance characteristics should be kept in mind and investigated in future studies.

### Limitations

Our study is limited by its monocentric design and the time restriction to one year. The data indicate that a multicentre approach could be useful. Second, some results are based on subjective participant ratings, so an objective evaluation could elucidate a different outcome. It could be significant that there is a high turnover of nursing staff, and that trained staff might have left the ward. A further limitation is that fewer staff members participated in the follow-up survey than in the surveys before, possibly due to staff turnover.

## Conclusions

In BC diagnostics in particular, a great deal of expertise is required in pre-analytics to obtain optimal results. These data show that there are considerable differences between individual departments in the same hospital, so that a "one size fits all" approach may not be realistically implementable. We were able to demonstrate that staff training in hygiene, handling, and logistics, with particular attention to interdisciplinary differences in prior knowledge, leads to a significant reduction in contamination in BCs. This effect can lead to more meaningful diagnostics and avoid unnecessary antibiotic therapies.

The implemented tools were evaluated differently with the best results for training. The additional use of the BD Vacutainer^®^ was not preferred due to subjectively assessed insufficient practicability compared to collection by syringe. Training without using the BD Vacutainer^®^ was also found to be effective. Overall, the direct user feedback provided valuable indications for improved practicability of the BC bottles in everyday clinical use, such as a predefined vacuum or optimised labelling. It shows that well established procedures should be evaluated regularly regarding correct implementation.

### Supplementary Information

Below is the link to the electronic supplementary material.

Supplementary file1 (DOCX 25 KB)

Supplementary file2 (DOCX 182 KB)

Supplementary file3 (DOCX 25 KB)

## Data Availability

Enquiries regarding the data can be made to the corresponding author.
