# Supplementary material for: A multi-pronged approach to improve blood culture diagnostics in different clinical departments: a single-centre experience
Source: Infection. 2023 Aug 17;52(1):183–95. doi: 10.1007/s15010-023-02083-y (PMC10810936; doi:10.1007/s15010-023-02083-y)
Supplement: Supplementary file 1 — Supplementary file1 (DOCX 25 KB) [file 15010_2023_2083_MOESM1_ESM.docx]

**Sup. 1: Questionnaire.**

A multi-pronged approach to improve blood culture diagnostics in different clinical departments: a single-centre experience.

Infection.

Elisabeth Neser; Philipp Jung; Alexander Halfmann; Matthias Schröder; Lorenz Thurner; Sören L. Becker; Sophie Schneitler.

Corresponding author: Dr. Sophie Schneitler, Institute of Medical Microbiology and Hygiene, Saarland University, E-mail address: [Sophie.Schneitler@uks.eu](mailto:Sophie.Schneitler@uks.eu).

**Questionnaire for blood culture sampling**

Gender:

- male female diverse

Age:

- 31-40 years
- >61 years
- 20-30 years
- 51-60 years
- <20 years
- 41-50 years

Your position at the ward:

- Nursing staff
- Physicians
- Ward secretary
- Nursing student
- Medical student
- Other (please specify): ________________________________

Who collects the blood cultures?

- Nursing staff
- Physicians
- Nursing staff and physicians
- Other (please specify): ________________________________

Who decides when and where blood cultures are collected?

- Nursing staff
- Physicians
- Nursing staff and physicians
- Other (please specify): ________________________________

Who generates the requests for blood culture diagnostics?

- Nursing staff
- Physicians
- Nursing staff and physicians
- Other (please specify): ________________________________

Your answers relate to the situation of a:

- Normal care unit
- Intensive care unit
- Outpatient clinic
- Other (please specify): ________________________________

**Please tick the correct answers to the questions below. Several answers may be correct.**

1. When are blood cultures collected?

- A) With fever
- B) Routinely on admission of the patient
- C) With chills
- D) With septic shock
- E) Routinely after invasive procedures

1. At which localisation are blood cultures collected?

- A) If a central catheter is available, a sampling should always be performed from this catheter
- B) If peripheral sampling is not possible, it can be performed from a central catheter
- C) From a central venous, arterial, or port catheter only if infection of this catheter is suspected
- D) peripherally in at least two localisations
- E) Peripheral sampling is not necessary if a central venous, arterial, or port catheter is available

1. How many blood cultures should be collected at what interval?

- A) One aerobic and one anaerobic blood culture is always sufficient
- B) One aerobic and one anaerobic blood culture at the same time, repeated sampling after 20 to 30 minutes
- C) An aerobic blood culture, after 20 to 30 minutes an anaerobic one
- D) Under certain circumstances, more often than twice in one day
- E) An aerobic blood culture is sufficient in most cases

1. How much blood should be filled in an aerobic or anaerobic blood culture bottle?

- A) Approximately 2 millilitres per blood culture bottle
- B) Approximately 5 millilitres per blood culture bottle
- C) Approximately 8 millilitres per blood culture bottle
- D) Approximately 20 millilitres per blood culture bottle
- E) The filling quantity does not differ for children and adults

1. What are different blood cultures used for?

- A) Different growth conditions for the bacteria
- B) Not all are suitable for blood, some are also made exclusively for other body fluids
- C) Fungi require a special blood culture bottle (special cultivation medium)
- D) If a nosocomial infection is suspected, special cultivation media are used
- E) Certain cultivation media can be examined immediately; others are put on hold for later examinations

1. In which order are blood cultures collected in case of conventional syringe collection?

- A) First aerobic, then anaerobic
- B) First anaerobic, then aerobic
- C) Two aerobic bottles
- D) Two anaerobic bottles
- E) The order is not important

1. Under what hygienic circumstances should the sampling be performed?

- A) Single disinfection of the skin is sufficient
- B) Disinfecting the blood culture bottles before inoculation
- C) Collection with sterile gloves
- D) Sterile blood collection is important to avoid contamination

E) As bacteria are probably detectable in the blood, disinfection can be dispensed with

1. What has to be considered when labelling the bottles?

- A) On each blood culture bottle, in addition to the patient label, the date and time of collection must be indicated
- B) The localisation of the blood sampling does not play a role in the labelling
- C) To be able to trace contamination, the person taking the sample should be noted
- D) It is recommended to indicate the sampling localisation, e.g., to better identify catheter infections
- E) Marking of the required filling quantity on the bottle facilitates collection

1. What has to be considered when collecting blood cultures from a central venous or port catheter?

- A) Draw and discard 5-10 millilitres of blood before drawing the blood for the blood cultures
- B) Disinfecting the catheter before blood collection
- C) After blood collection, the central venous or port catheter should be rinsed
- D) The collection must be done from each catheter segment
- E) Blood should only be collected from a port catheter in special cases

1. What has to be considered after the blood culture sampling?

- A) Immediate transport in a thermal container to the laboratory
- B) Immediate transport to the laboratory by pneumatic tube
- C) If it is expected that further patient samples will be collected, it is not a problem to remain on the ward for up to one hour.
- D) If the blood cultures are stored in the refrigerator, they can remain on the ward until the next day
- E) If the blood cultures are stored in the refrigerator, they can remain on the ward until the next day
