# Supplementary material for: A multi-pronged approach to improve blood culture diagnostics in different clinical departments: a single-centre experience
Source: Infection. 2023 Aug 17;52(1):183–95. doi: 10.1007/s15010-023-02083-y (PMC10810936; doi:10.1007/s15010-023-02083-y)

**Sup. 2: Information card**

A multi-pronged approach to improve blood culture diagnostics in different clinical departments: a single-centre experience.

Infection.

Elisabeth Neser; Philipp Jung; Alexander Halfmann; Matthias Schröder; Lorenz Thurner; Sören L. Becker; Sophie Schneitler.

Corresponding author: Dr. Sophie Schneitler, Institute of Medical Microbiology and Hygiene, Saarland University, E-mail address: [Sophie.Schneitler@uks.eu](mailto:Sophie.Schneitler@uks.eu).


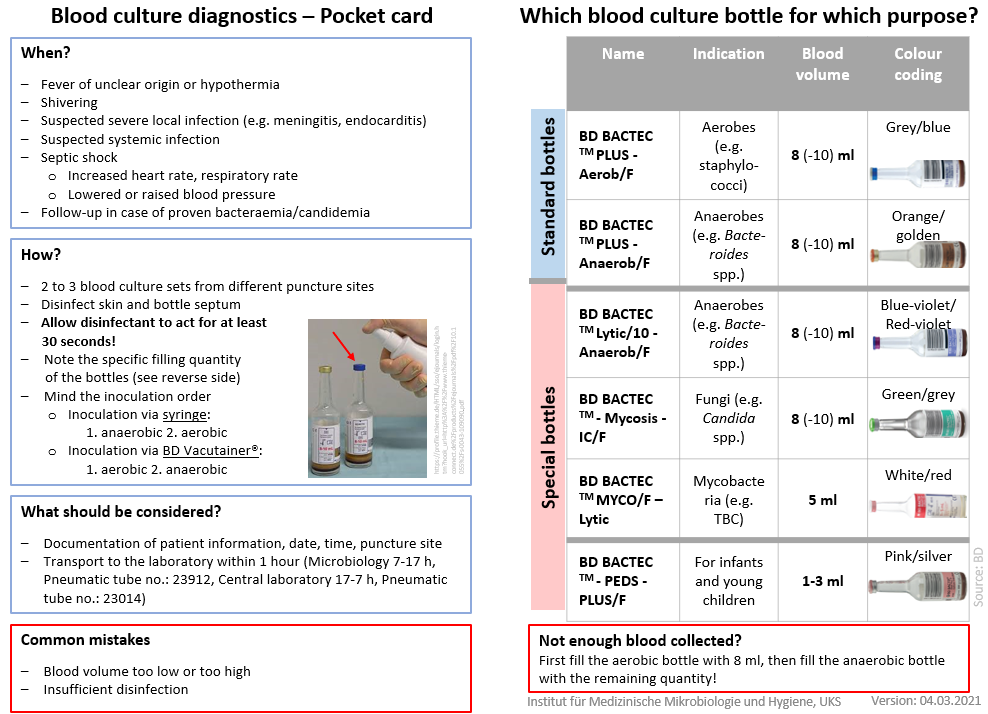

Supplement: Supplementary file 2 — Supplementary file2 (DOCX 182 KB) [file 15010_2023_2083_MOESM2_ESM.docx]
